# Supplementary material for: Resolving the full spectrum of human genome variation using Linked-Reads
Source: Genome Res. 2019 Apr;29(4):635–45. doi: 10.1101/gr.234443.118 (PMC6442396; doi:10.1101/gr.234443.118)
Supplement: Supplemental Material [file supp_gr.234443.118_Supplemental_longranger-2.2.zip › longranger-2.2.tar.bz2!/lr2.2-public/lib/stan/cmdstan/stan/doc/api/html/dir_22b468d2bc5e21cba3741edb912cd36a.html]

Stan: src/stan/math/error\_handling/matrix Directory Reference


|  |  |
| --- | --- |
|  | Stan  2.5.0  probability, sampling & optimization |


- Main Page
- Related Pages
- Namespaces
- Classes
- Files

All Classes Namespaces Files Functions Variables Typedefs Enumerations Enumerator Friends Macros Pages

- src
- stan
- math
- error\_handling
- matrix

matrix Directory Reference

|  |  |
| --- | --- |
| Files | |
| file | check\_cholesky\_factor.hpp [code] |
|  | |
| file | check\_cholesky\_factor\_corr.hpp [code] |
|  | |
| file | check\_column\_index.hpp [code] |
|  | |
| file | check\_corr\_matrix.hpp [code] |
|  | |
| file | check\_cov\_matrix.hpp [code] |
|  | |
| file | check\_ldlt\_factor.hpp [code] |
|  | |
| file | check\_lower\_triangular.hpp [code] |
|  | |
| file | check\_matching\_dims.hpp [code] |
|  | |
| file | check\_matching\_sizes.hpp [code] |
|  | |
| file | check\_multiplicable.hpp [code] |
|  | |
| file | check\_nonzero\_size.hpp [code] |
|  | |
| file | check\_ordered.hpp [code] |
|  | |
| file | check\_pos\_definite.hpp [code] |
|  | |
| file | check\_pos\_semidefinite.hpp [code] |
|  | |
| file | check\_positive\_ordered.hpp [code] |
|  | |
| file | check\_range.hpp [code] |
|  | |
| file | check\_row\_index.hpp [code] |
|  | |
| file | check\_simplex.hpp [code] |
|  | |
| file | check\_size\_match.hpp [code] |
|  | |
| file | check\_spsd\_matrix.hpp [code] |
|  | |
| file | check\_square.hpp [code] |
|  | |
| file | check\_std\_vector\_index.hpp [code] |
|  | |
| file | check\_symmetric.hpp [code] |
|  | |
| file | check\_unit\_vector.hpp [code] |
|  | |
| file | check\_vector.hpp [code] |
|  | |
| file | constraint\_tolerance.hpp [code] |
|  | |
| file | validate\_non\_negative\_index.hpp [code] |
|  | |


---

[ Stan Home Page ]

*© 2011–2014,
Stan Development Team.*
